# Identification of Leaf Proteins Differentially Accumulated between Wheat Cultivars Distinct in Their Levels of Drought Tolerance

**DOI:** 10.1371/journal.pone.0125302

**Published:** 2015-05-18

**Authors:** Zhiwei Cheng, Kun Dong, Pei Ge, Yanwei Bian, Liwei Dong, Xiong Deng, Xiaohui Li, Yueming Yan

**Affiliations:** 1 College of Life Science, Capital Normal University, 100048 Beijing, China; 2 Hubei Collaborative Innovation Center for Grain Industry (HCICGI), 434025 Jingzhou, China; Institute of Genetics and Developmental Biology, Chinese Academy of Sciences, CHINA

## Abstract

The drought-tolerant ‘Ningchun 47’ (NC47) and drought-sensitive ‘Chinese Spring’ (CS) wheat (*Triticum aestivum* L.) cultivars were treated with different PEG6000 concentrations at the three-leaf stage. An analysis on the physiological and proteomic changes of wheat seedling in response to drought stress was performed. In total, 146 differentially accumulated protein (DAP) spots were separated and recognised using two-dimensional gel electrophoresis. In total, 101 DAP spots representing 77 unique proteins were identified by matrix-assisted laser desorption/ionisation time-of-flight mass spectrometry. These proteins were allocated to 10 groups according to putative functions, which were mainly involved in carbon metabolism (23.4%), photosynthesis/respiration (22.1%) and stress/defence/detoxification (18.2%). Some drought stress-related proteins in NC47, such as enolase, 6-phosphogluconate dehydrogenase, Oxygen-evolving enhancer protein 2, fibrillin-like protein, 2-Cys peroxiredoxin BAS1 and 70-kDa heat shock protein, were more upregulated than those in CS. Multivariate principal components analysis revealed obvious differences between the control and treatments in both NC47 and CS, while cluster analysis showed that the DAPs displayed five and six accumulation patterns in NC47 and CS, respectively. Protein–protein interaction network analysis showed that some key DAPs, such as 2-Cys peroxiredoxin BAS1, RuBisCO large subunit-binding protein, 50S ribosomal protein L1, 6-phosphogluconate dehydrogenase, glyceraldehyde 3-phosphate dehydrogenase isoenzyme and 70-kDa heat shock protein, with upregulated accumulation in NC47, had complex interactions with other proteins related to amino acid metabolism, carbon metabolism, energy pathway, signal transduction, stress/defence/detoxification, protein folding and nucleotide metabolism. These proteins could play important roles in drought-stress tolerance and contribute to the relatively stronger drought tolerance of NC47.

## Introduction

Global warming is caused by climate changes that may increase abiotic stresses on crop production [1]. In particular, water availability is a major limitation for plant production and affects the distribution of plant species [2]. Then, drought stress frequently occurs during the seedling stage, especially at the early stages, and deleteriously affects crop growth and reduces grain yield worldwide [3]. Wheat (*Triticum aestivum* L.) is an important grain crop cultivated worldwide due to its excellent yield and nutritional value. Like many other crop species, wheat production is largely affected by drought stress. Thus, elucidating the molecular mechanisms of wheat seedling survival under drought stress and the adaptive proteins they use in response to adverse growing conditions is essential [4].

Plant response to drought stress is complex, involving both morphological and biochemical changes [5]. Drought and salt stress have an osmotic effect on crop growth and photosynthesis. Deleterious effects of drought on photosynthesis will be mediated by the responsiveness of (i) the respiration system, electron transport and ATP synthesis in the mitochondria; (ii) the accumulation of stress metabolites; and (iii) gene expression and protein synthesis [6]. The response to drought normally involves a mixture of stress avoidance and tolerance responses that vary with different genotypes [7]. Then, the most severe effect of drought is impaired germination and poor seedling establishment [8–9]. Therefore, many proteins related to stress/defence/detoxification, carbohydrate metabolism and photosynthesis are involved in the process [10]. In addition, plants can withstand water-deficit conditions by decreasing energy expenditure, increasing ATP production and reducing oxidative damage [11]. Concurrently, plant osmolytes can also act as free radicals and reactive oxygen species (ROS) scavengers, or as chemical chaperones by directly stabilising membranes and/or proteins and maintaining protein structure and function [12].

Bread wheat is an allopolyploid, with three homeologous sets of seven chromosomes in each of the A, B and D subgenomes, which are each ~5.5 Gb in size. Therefore, the response of wheat to drought stress has been widely studied using transcriptome, proteome and metabolome analyses. In modern wheat, comparisons of transcriptomes have revealed several unique genes or expression patterns, such as differential usage of inositol trisphosphate (IP3)-dependent signal transduction pathways, and ethylene- and abscisic acid (ABA)-dependent signalling [13]. However, transcriptome analysis can dictate protein abundance, and differential expression of the two macromolecules is not always well correlated [14]. Proteomic approaches can provide information missing from DNA or mRNA analyses in that they focus on the actively translated portion of the genome.

Stress resistance is conferred by proteins that function in stress signalling, transcription regulation, cellular detoxification, protection of macromolecules and other processes [15]. At the metabolite level, many compounds are increased to provide osmoprotective functions, prevent dissociation of enzymes and decrease the number of ROS present in the cell. Levels of amino acids, most notably proline, tryptophan and the branched chain amino acids leucine, isoleucine and valine, increase in bread wheat in response to drought stress [16]. Studies on cereal crops have revealed several novel genes, proteins and metabolites, as well as pathways previously not known to play a part in drought stress [4]. Nevertheless, the influence of drought stress on cereal crop growth, development and metabolism has been studied extensively. It is still not enough to understanding the drought tolerance mechanisms of wheat which have a large and complex genome and transcriptome.

Recently, high-throughput, low-cost sequencing technologies have been applied to assemble the gene space of *Triticum urartu* [17] and *Aegilops tauschii* [18], two diploid species related to bread wheat. The 3B chromosome of bread wheat has been ordered and structured [19], which contributes to the crop proteomic research on the response of wheat to various biotic and abiotic stresses.

In this study, we performed an in-depth analysis of the physiological and proteomic changes of seedling leaves under drought stress. The study involved two Chinese spring wheat cultivars: drought-tolerant ‘Ningchun 47’ (NC47) and drought-sensitive ‘Chinese Spring’ (CS). Our results revealed the central metabolic changes of seedling leaves involved in drought tolerance and adaptation, and provide new insights into the proteomic mechanisms of the plant drought response.

## Materials and Methods

### Plant materials and drought treatments

The materials used in this study included two Chinese spring wheat cultivars (*Triticum aestivum* L.): drought-sensitive Chinese Spring (CS) and drought-tolerant Ningchun 47 (NC47) developed in Ningxia Academy of Agriculture and Forestry Sciences [20]. The seeds were surface sterilized by 5% sodium hypochlorite for 5 min, and rinsed 4 times in sterile distilled water. Then, wheat seeds were germinated in the dark condition for 48 h at 25°C before transplanted into nutrient solution. The seedlings were grown in a growth chamber under 26/18°C (16-h day/8-h night) and relative humidity of 60–75%. To provide whole nutrition for wheat seedlings, Hogland solution was changed every 2 days, which consists of 5 mM KNO_3_, 2 mM MgSO_4_, 1 mM KH_2_PO_4_, 5 mM Ca(NO_3_)_2_, 50 μM FeNa_2_(EDTA)_2_, 50 μM H_3_BO_3_, 10 μM MnC1_2_, 0.8 μM ZnSO_4_, 0.4 μM CuSO_4_ and 0.02 μM (NH_4_)_6_MoO_24_). The seedlings at three-leaf stage were treated with five different PEG6000 concentrations (0, 15%, 20%, 25%, and 30%) in three biological replicates. After 48 h treatment, the seedling leaves were harvested, and the leaf physiological parameters including the relative water content (RWC), soluble sugar and proline content were measured immediately after sampling. The remaining leaf samples were kept frozen in -80°C for later protein extraction and proteome analysis.

### Measurement of leaf physiological parameters

#### RWC

Fresh weight of seedling leaves was weighted immediately after harvesting while dry weight was measured after drying the leaves in an oven at 80°C for three days. Leaf RWC was calculated by the following formula:

RWC=[(leaf fresh weight-leaf dry weight)/leaf fresh weight]×100%.

#### Soluble sugar content

Leaf soluble sugar content was determined according to Zhang et al [21]. Leaf samples (1 g fresh weight) were homogenized in 2 ml 10% trichloroacetic acid (TCA) and then 8 ml 10% TCA was added for further grinding and centrifuged for 10 min at 5,000g. A volume of 1 ml of supernatant sample was combined with 2 ml 0.6% thiobarbituric acid (TBA) and incubated in boiling water for 15 min, and then quickly cooled in an ice bath. The mixture was centrifuged at 10,000 g for 5 min and the absorbance of supernatant was monitored at 532, 600 and 450 nm. The content of soluble sugars (C_1_) (mmol L^-1^) was calculated by the following formulas (D450 represents the absorbance in the wavelengths of 450): C_1_ = 11.71×D450.

#### Free proline content

Leaf free proline content was measured according to Bates et al. [22] with some modifications. Approximately 0.5 g of samples was homogenized with 5 ml of 3% (w/v) aqueous sulfosalicylic acid solution. The homogenate was centrifuged at 3000 g for 20 min. The supernatant, acid–ninhydrin agent and glacial acetic acid (2 mL each) were mixed and boiled for 1 h. The reaction mixture was extracted with 4 mL toluene. Then the homogenate was centrifuged at 3000 g for 10 min. The absorbance at 520 nm was determined using L-proline as a standard. Proline contents were expressed in micrograms per gram per unit fresh weight.

### Protein extraction, 2-DE and image analysis

Proteins from three biological replicates in each treatment were extracted using the phenol extraction method [10]. Each sample was extracted three times. First-dimensional electrophoresis was performed on an IPGphor IEF (isoelectric focusing) system. 1000 μg extracted protein was diluted with an IEF rehydration buffer (7 M urea, 2 M thiourea, 2% w/v CHAPS, 0.004% bromphenol blue) containing 1% DTT and 0.5% IPG buffer to get the final volume to 360 μl for IEF. Then 360 μl protein solution was loaded onto a commercially available precast IPG strip with an 18-cm linear pH 4–7 gradient and rehydrated at 30 V for 12 h, and IEF was performed on the IPGphor apparatus. The focusing conditions for first-dimension IEF was under the following conditions: 300 V for 1 h, 500 V for 1 h, 1000 V for 1 h, 3000 V for 1 h, and 8000 V to achieving 65,000 V-hr. Before the SDS-PAGE, the strips were equilibrated for 15 min in 5 ml of equilibration buffer (6 M urea, 50 mM Tris-HCl at pH 8.8, 30% (v/v) glycerol, 2% (w/v) SDS, 0.01% bromophenol blue and 1% (w/v) DTT). The second equilibration treatments were equilibrated for 15 min in 5 ml equilibration buffer containing 4% (w/v) iodoacetamide instead of 1% DTT. The strips were placed on the top of vertical on 12% SDS-polyacrylamide self-cast gels by using a Protean II Multi Cell apparatus. The electrophoresis was carried out at 25°C and 10 A/gel for 30 min and then at 25 A/gel until the dye front reached to the bottom of the gel. After electrophoresis, protein spots were visualized by colloidal Coomassie Brilliant Blue (CBB) staining (R-250/G-250 = 4:1), and destained by the solution containing 10% ethanol and 10% acetic acid.

The 2-DE images were scanned by GS-800 Calibrated Densitometer and statistical analysis was performed by the ImageMaster^TM^ 2-D platinum software version 7.0, which allowed spot detection, landmarks identification, aligning/matching of spots within gels, quantification of matched spots according to the manufacturer’s instructions. Further manual editing was performed to correct the mismatched and unmatched spots. Spot detection parameters were set as follows: smooth 6, min area 4 and saliency 6. Spot volume was used as the analysis parameter for quantifying protein accumulation amount. Relative spot volumes (% V) (V = integration of OD over the spot area; %V = V single spot/V total spot) were used for quantitative analysis in order to decrease experimental errors. The normalized volume of spots on three replicate 2-D gels was averaged and standard deviation was calculated for each condition. Spot volume ratios that showed a statistically significant difference (≥ two-fold difference in vol. %; *p* < 0.05) by Student’s t-test at one or more drought treatments were processed for further analysis.

### In-gel digestion, MALDI-TOF/TOF-MS and database searching

Protein spots showing significant changes in abundance during the treatments were selected for protein identification from the CBB blue staining gels. Tryptic peptides were analyzed with a MALDI-TOF/TOF mass spectrometer. Then, unidentified spots were further analyzed using a MALDI-TOF/TOF mass 4800 Proteomics Analyzer, and searched in the NCBI non-redundant green plant database using GPS Explorer software version 2.0 (Applied Biosystem) [10]. The tryptic spectra mass range is from 800 to 4000 Da, the peptide mass tolerance and fragment mass tolerance were ± 0.2 Da, and allowed one missed cleavage, carbamidomethyl (Cys) and oxidation (Met) were specified as variable modifications. Only proteins with protein score CI >95% and total ion score CI >95% were considered to be credibly identified.

### Multivariate and cluster analysis

The multivariate method used was Principal Component Analysis (PCA) performed in SPSS (version 19.0). PCA is a way of identifying patterns in data, and expressing the data in such a way as to emphasis their similarities and differences. It can compress the data, that is, by reducing the number of dimensions without much loss of information based on their similarities and differences, and define a limited number of “principal components” which describe independent variation structures in the data [23]. The loading plot of PCA was displayed with the average vol. % of DAP spots. Therefore, PCA can indicate relationships among groups of variables in a data set and show relationships that might exist between objects.

Cluster analysis of the DAP spots data was performed by employing the Euclidean distance method over a complete linkage dissimilarity matrix using the Cluster 3.0 and TreeView.

### Bioinformatics

Subcellular locations of DAPs were predicted based on the combination of WoLF PSORT (http://wolfpsort.org/) [24] UniprotKB (http://www.uniprot.org/).

The sequences of all the DAPs were used for BLAST analysis with the National Center for Biotechnology Information (NCBI) clusters of Eukaryotic Orthologous Groups (KOG) database to obtain the KOG numbers of those proteins by eggNOG (http://eggnog.embl.de/version_3.0/). A data set containing all the KOG numbers was then used for protein-protein interactions (PPI) analysis by using the Search Tool for Retrieval of Interacting Genes/Proteins (STRING) database (version 9.1, http://string-db.org) [20,25,66]. Only the interactions that had a confidence score of at least 0.7 and were exclusively based on coexpression and experiment conditions were used to construct the network, then they were displayed using the Cytoscape (version 3.0.0) software [20,26]. Furthermore, a putative metabolic pathway has been gained using KEGG pathway (www.genome.jp/kegg/pathway.html) databases.

## Results

### Morphological and physiological response to drought stress in wheat seedling leaves

**Fig 1:**
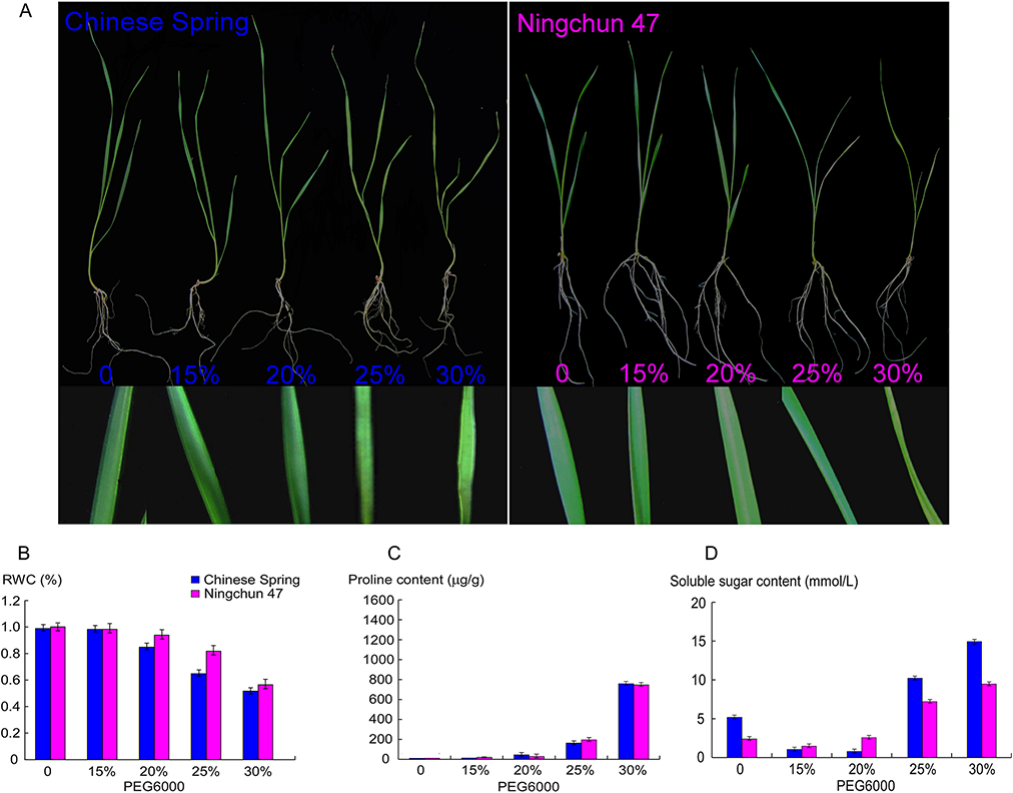
Morphological and physiological changes in seedlings of two wheat (*Triticum aestivum* L.) cultivars, ‘Chinese Spring’ (CS) and ‘Ningchun 47’ (NC47), during 48 h of PEG-mediated drought stress. (A) Morphological changes of wheat seedlings in response to different PEG6000 concentration treatments. (B) Leaf relative water content (RWC) analysis. (C) Proline content analysis. (D) Soluble sugar content analysis.

Under the exogenous and different concentration gradients of polyethylene glycol 6000 (PEG6000)-induced stress, the morphological and physiological characteristics of wheat seedling leaves changed significantly. As shown in Fig 1A, differences in the width and colour of second leaves became more obvious as the PEG6000 concentration increased. Meanwhile, the inner surface of leaves curled and wilted, and the leaves curled into a cylindrical shape when the PEG6000 concentration reached 30%. The concentration gradient of PEG6000 had a distinct influence on the growth of the third leaf, except for the 15% PEG6000 concentration. Similar results were also observed in the seedling leaves of NC47, but the degree of leaf curling and wilting of NC47 was not as severe as that for CS at 25% and 30% PEG6000 concentrations.

To further clarify the effects of the PEG6000 concentration on wheat seedling growth, we measured changes in three physiological indices of seedling leaves, including the relative water content (RWC), and soluble sugar and proline content. Compared to the control, the RWC of both spring wheat cultivars decreased gradually as the PEG6000 concentration increased (Fig 1B). At a 15% PEG6000 concentration, the RWC of treated-wheat seedlings displayed no detectable changes compared with the control. When the PEG6000 concentration reached 20% and 30%, the RWC of CS seedling leaves began to decline. This reduction in the RWC was more obvious for CS than NC47. According with previous research, the rate of RWC in plants with high resistance against drought is higher than others [27], indicating that NC47 has greater tolerance to drought stress than CS. The proline content of both cultivars displayed a similar trend, with few changes at 15–25% PEG6000, but a dramatic increase at 30% PEG6000 (Fig 1C). Note that the soluble sugar content of CS at a 15% and 20% PEG6000 concentration significantly decreased, but no obvious changes occurred in the NC47 soluble sugar content. When the PEG6000 concentration reached 25% and 30%, the soluble sugar content increased substantially in both cultivars (Fig 1D).

### Differentially accumulated protein (DAP) identification

**Fig 2:**
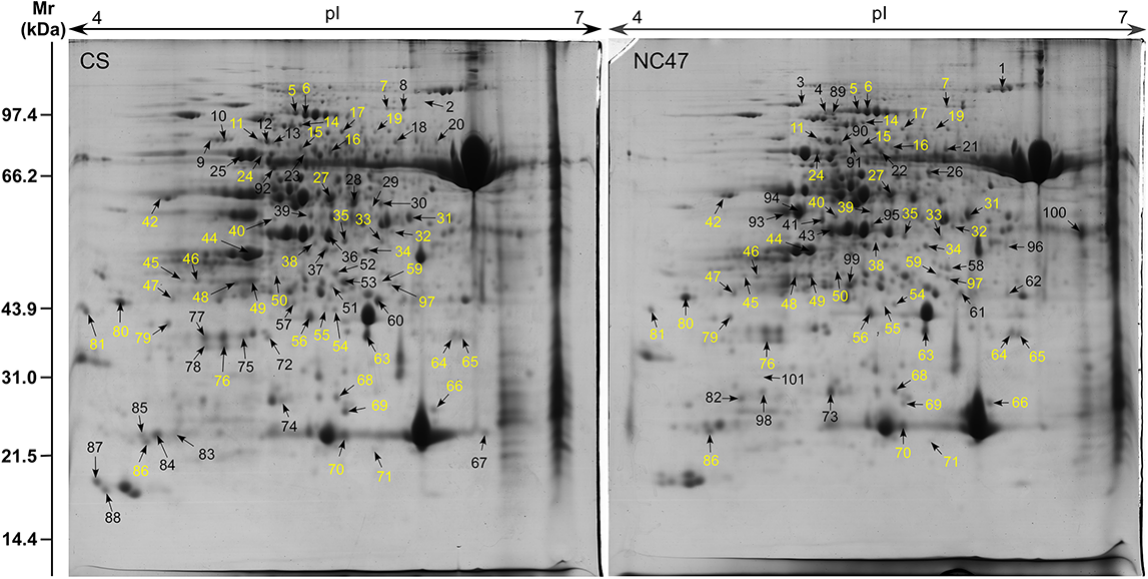
Proteome maps of two spring wheat varieties. (A) CS under the control condition. (B) NC47 under the control condition. The numbered protein spots were identified by MALDI-TOF/TOF, and yellow numbers represent the common DAP spots in NC47 and CS.

The proteome maps of seedling leaves from CS and NC47 grown in five different PEG6000 concentrations were produced from two-dimensional gel electrophoresis (2-DE), as shown in Fig 2. In general, both cultivars displayed similar 2-DE patterns. The protein spots were distributed uniformly from pH 4 to 7, and their molecular masses ranged from 10 to 100 kDa. Most of the protein spots were located in the high-molecular-mass region (30–90 kDa). More than 1000 protein spots were detected in all gels, of which 146 DAP spots with at least twofold differences were selected for MALDI-TOF/TOF mass spectrometer identification. Finally, 101 different DAP spots were identified with a high degree of confidence. These DAP spots represented 77 unique proteins of which 62 and 57 DAPs were from CS and NC47, respectively, and 38 DAPs were common to both cultivars (Fig 2). All DAPs with three times repetition are listed in S1 Table, and detailed MS information is provided in S2 Table.

**Fig 3:**
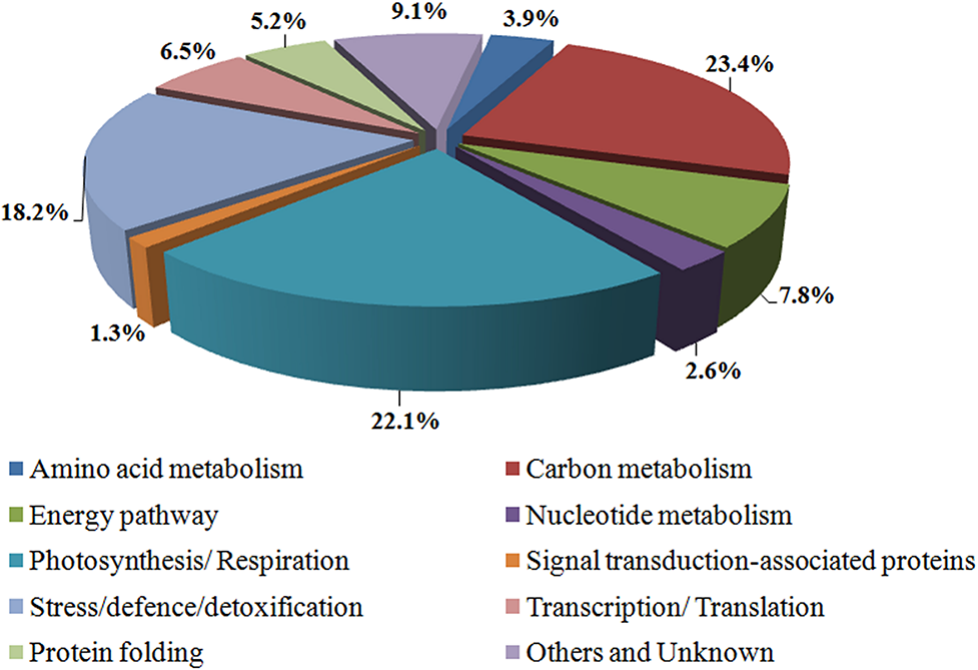
Functional distribution of identified differentially accumulated proteins in CS and NC47.

According to the UniProt and AgBase website (version 2.00, http://www.agbase.msstate.edu/), 77 unique proteins were classified into 10 functional categories: carbon metabolism (23.4%), photosynthesis/respiration (22.1%), stress/defence/detoxification (18.2%), energy pathway (7.8%), transcription/translation (6.5%), protein folding (5.2%), amino acid metabolism (3.9%), nucleotide metabolism (2.6%), signal transduction (1.3%) and unknown (9.1%), as shown in Fig 3. The last category included eight identified proteins (spots 31, 37, 46, 47, 53, 59, 72 and 88) annotated as either unknown or without function. The subcellular localisation of 101 identified DAPs was predicted using WoLF PSORT and UniprotKB. The proteins covered a wide range of subcellular locations, but they were mainly in plastids, the cytoplasm and plasma membrane (S1 Table).

### Multivariate and cluster analysis

**Fig 4:**
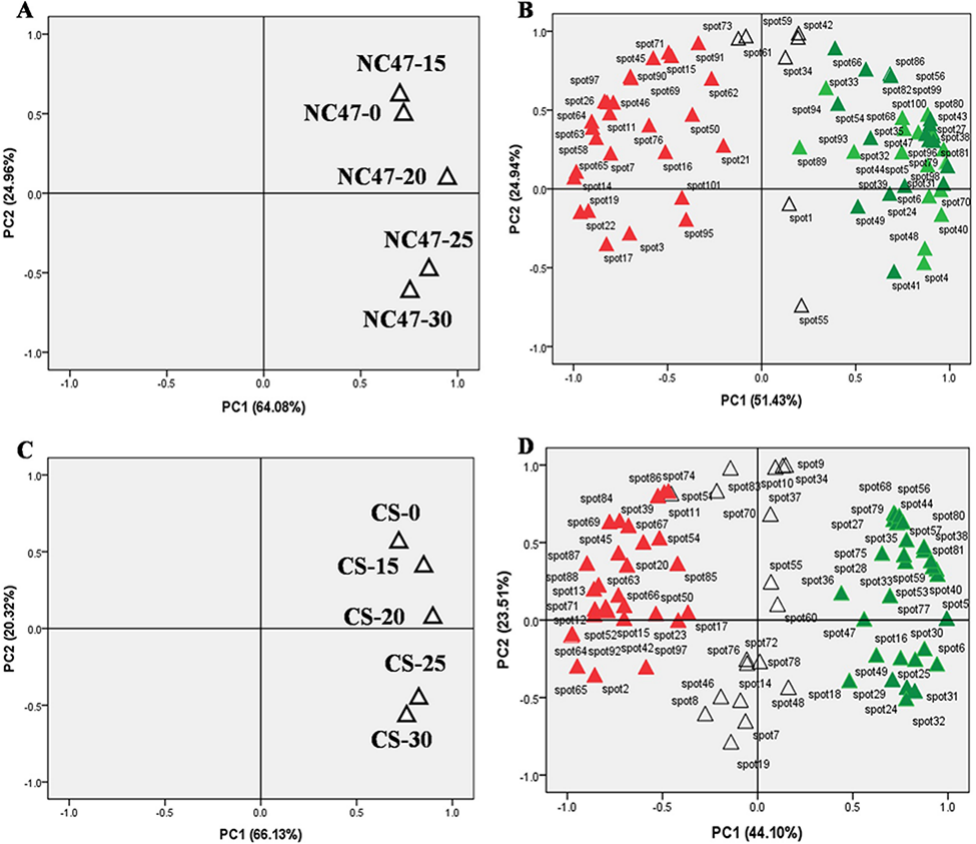
Principal components analysis (PCA) of the set of 101 DAP spots in NC47 and CS. (A) PCA of individual experimental samples in NC47. (B) PCA of 69 differentially accumulated protein spots in NC47. (C) PCA of individual experimental samples in CS. (D) PCA of 77 differentially accumulated protein spots in CS. Red spots indicate an upregulated protein; green spots indicate a downregulated protein. CS-0, CS-15, CS-20, CS-25 and CS-30 represent the 0, 15%, 20%, 25% and 30%, respectively, of the PEG6000 concentration treatment in CS. NC47-0, NC47-15, NC47-20, NC47-25 and NC47-30 represent the 0, 15%, 20%, 25% and 30%, respectively, of the PEG6000 concentration treatment in NC47.

Principal components analysis (PCA) is an exploratory tool often used to obtain a multidimensional overview of multivariate data, such as the spot volumes of digitised 2-DE gels. PCA can reveal hidden structures present in the data and thus enables the identification of potential outliers and clusters [28]. In this study, data reduction was applied to the entire set of differential spots (*n* = 101) using PCA. Applying the set of 69 DAP spots in NC47, PCA revealed major differences in protein abundance patterns when comparing the control and PEG6000 concentrations. As shown in Fig 4, the control (NC47-0) and PEG6000-treated samples (NC47-15, NC47-20, NC47-25 and NC47-30) were grouped differently in PCA plots (Fig 4A), indicating that NC47-0 had no obvious differences compared with NC47-15. Conversely, an obvious difference was observed between the control sample (NC47-0) and high PEG6000-treated samples (NC47-25 and NC47-30). Furthermore, the upregulated (red) and downregulated (green) DAP spots, which were present in the NC47 control and at different PEG6000 concentration treatments, were located in clearly different parts of the PCA plot (Fig 4B). The upregulated DAPs (Fig 4B, left) were opposite to downregulated DAPs (Fig 4B, right). Similar results were also observed in the CS treatments (Fig 4C and 4D). PCA revealed obvious differences in patterns of relative protein abundance, which was verified by subsequent cluster analysis.

**Fig 5:**
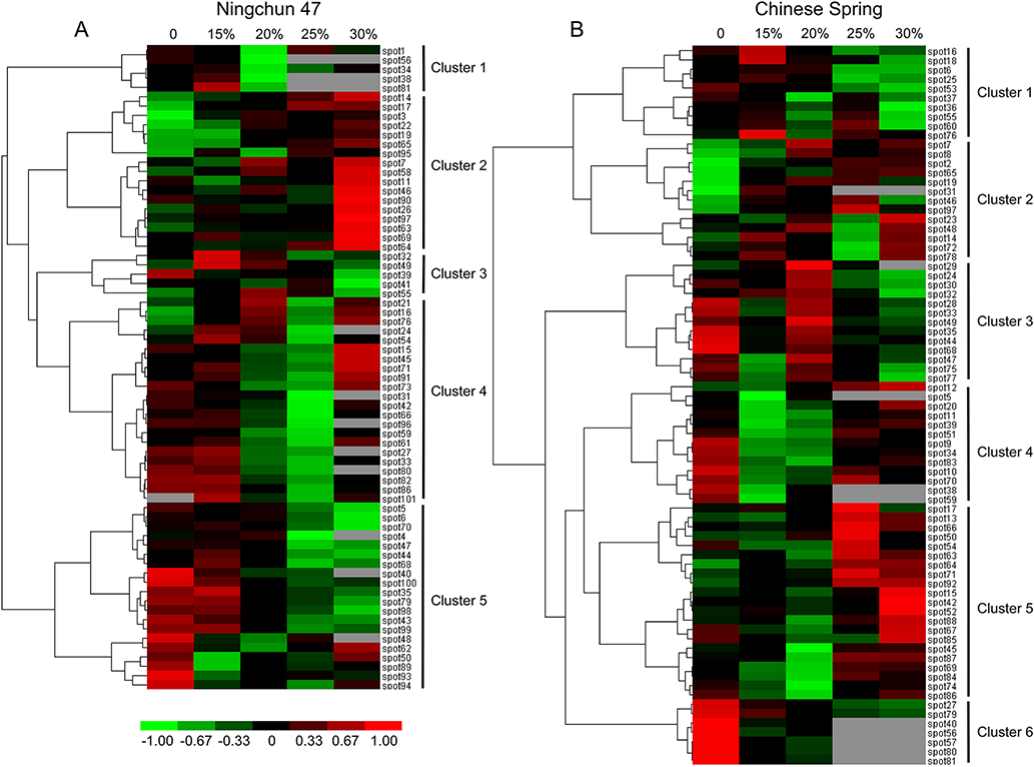
Hierarchical cluster analysis of DAP spots in NC47 and CS. Red colour indicates a positive abundance in protein spots; green colour denotes a negative abundance in protein spots. (A) Cluster analysis of 69 differentially accumulated proteins in NC47. (B) Cluster analysis of 77 differentially accumulated proteins in CS. 0, 15%, 20%, 25% and 30% represent the PEG6000 concentration treatment.

For cluster analysis, the relative ratios of DAP spots of the proteome dataset listed in File S1 were used to define similarity and complete linkage. Cluster analysis of the differential spots was performed using the Euclidean distance method over a complete linkage dissimilarity matrix (Fig 5). The results showed that the 69 DAP spots in NC47 (Fig 5A) were divided into five accumulation patterns (Clusters 1–5) according to hierarchical clustering algorithms. Cluster 1 mainly included photosynthesis/respiration proteins, which displayed a decreased abundance under 20% PEG6000-induced stress. Cluster 2 exhibited an increased protein abundance under a high PEG6000 (25% and 30%) concentration and mainly included carbon metabolism-related proteins (41.2%), transcription/ translation-related proteins (11.8%), protein folding-related proteins (11.8%) and photosynthesis/respiration proteins (11.8%). Cluster 3 also mainly included photosynthesis/respiration proteins, but it exhibited a relative reduction under a 30% PEG6000 concentration. Cluster 4 mainly included stress/defence/ detoxification-related proteins (36.2%), carbon metabolism-related proteins (22.7%) and photosynthesis/respiration proteins (18.2%), which displayed high protein accumulation levels under the control and low PEG6000 (15%) concentration, and showed reductions under a 25% PEG6000 concentration. Cluster 5 displayed the highest protein accumulation level for the control group, which mainly included stress/defence/detoxification-related proteins (25.0%), carbon metabolism-related proteins (25.0%), photosynthesis/respiration proteins (15.0%), amino acid metabolism-related proteins (10%) and energy pathway-related proteins (10%). The 77 DAP spots in the CS (Fig 5B) treatment group were allocated to six accumulation patterns (Clusters 1–6). The main accumulation pattern differences occurred in Cluster 2, which displayed no obvious regularity in CS, and Cluster 3, which displayed high protein accumulation levels under the 20% PEG6000 concentration.

### Comparative proteome analysis of NC47 and CS response to drought stress

For wheat, drought stress occurs globally and often during the early seedling stage. Consequently, changes in seedling leaves are often used as an indicator of drought stress. As we all know the main physiological functions of seedling leaves are photosynthesis and carbon metabolism. Therefore, the accumulation levels of DAPs involved in photosynthesis and carbon metabolism are significantly altered under drought stress. In addition, stress/defence/ detoxification-related proteins are also greatly affected (Table 1, and more details in S1 Table).

**Table 1:** Comparison of some important DAPs in seedling leaves of NC47 and CS.

Spot no.	Protein name	Accession no.	Predicted subcellular localisation^a^	Average volume ratio CS0:15%:20%:25%:30%	Average volume ratio NC470:15%:20%:25%:30%
**Amino acid metabolism**					
5	Methionine synthase 1 enzyme	gi|68655495	Cyto	1: 0.69: 1.02: 0.00: 0.00	1: 0.60: 0.69: 0.26: 0.13
**Carbon metabolism**					
7	Cytosolic aconitase	gi|290783890	Cyto	1: 1.51: 3.94: 1.97: 2.76	1: 0.80: 1.51: 1.04: 1.76
16	Phosphoglycerate mutase	gi|32400802	Cyto	1: 1.36: 0.90: 0.65: 0.74	1: 1.52: 2.05: 1.09: 1.97
17	NADP-dependent malic enzyme	gi|158701881	P	1: 1.12: 1.03: 1.83: 0.88	1: 1.70: 1.68: 2.43: 2.31
19	NADP-dependent malic enzyme	gi|158701881	P	1: 2.41: 3.34: 3.04: 1.98	1: 0.97: 2.35: 2.38: 3.14
22	Enolase	gi|90110845	Cyto	N^b^	1: 1.30: 2.04: 1.83: 2.33
26	6-phosphogluconate dehydrogenase, decarboxylating	gi|357110692	Mito	N	1: 1.77: 1.22: 1.51: 4.91
40	Chloroplast fructose-bisphosphate aldolase	gi|223018643	P	1: 0.58: 0.60: 0.00: 0.00	1: 0.72: 0.59: 0.56: 0.00
45	Glyceraldehyde 3-phosphate dehydrogenase	gi|15222111	Cyto	1: 1.06: 0.56: 1.15: 1.21	1: 1.00: 0.82: 0.63: 1.79
90	β-amylase	gi|3334120	P	N	1: 0.83: 0.86: 0.75: 1.73
97	Triosephosphate-isomerase	gi|11124572	Cyto	1:1.44: 1.41: 2.10: 1.36	1: 1.19: 1.13: 1.13: 2.58
**Energy pathway**					
14	vacuolar proton-ATPase subunit A	gi|90025017	chloroplast	1: 1.77: 1.28: 0.81: 1.71	1: 1.22: 1.39: 1.65: 2.24
**Nucleotide metabolism**					
58	Chitinase 2	gi|18146827	chloroplast	N	1: 1.23: 1.51: 1.23: 2.01
**Photosynthesis/ Respiration**					
64	Oxygen-evolving enhancer protein 2	gi|131394	P	1: 1.79: 1.35: 3.23: 3.05	1: 0.83: 0.90: 1.53: 3.21
65	Oxygen-evolving enhancer protein 2	gi|131394	P	1: 4.15: 2.79: 6.07: 7.04	1: 1.16: 3.45: 4.65: 8.18
93	Phosphoribulokinase	gi|125580	P	N	1: 0.21: 0.34: 0.35: 0.29
**Stress/defence/detoxification**					
33	Dehydroascorbate reductase	gi|28192421	Mito	1: 0.20: 0.89: 0.33: 0.17	1: 1.24: 0.46: 0.32: 0.74
62	Ascorbate peroxidase	gi|3688398	Cyto	N	1: 0.61: 0.42: 0.66: 1.06
76	2-Cys peroxiredoxin BAS1	gi|2829687	P	1: 1.89: 0.83: 1.23: 1.06	1: 1.52: 2.05: 0.97: 2.31
95	Fibrillin-like protein	gi|29367475	P	N	1: 1.83: 0.99: 2.03: 1.72
**Transcription/Translation**					
63	Predicted protein	gi|326490946	P	1: 1.08: 0.76: 1.89: 1.37	1: 1.25: 1.32: 1.37: 2.67
69	50S ribosomal protein L1	gi|195638036	Nucl	1: 0.70: 0.53: 1.23: 1.18	1: 1.31: 0.90: 0.90: 2.46
**Protein folding**					
3	70-kDa heat shock protein	gi|254211611	P	N	1: 2.12: 2.70: 2.32: 2.63
11	RuBisCO large subunit-binding protein subunit	gi|2493650	P	1: 0.47: 0.57: 0.93: 1.03	1: 0.66: 0.89: 0.92: 1.62

^a^Cyto, cytoplasm; P: plastid; Mito: mitochondria; Nucl: nuclear; PM: Plasma membrane.

^b^"N" represent the DAP spot was no obvious difference (< 2.0-fold).

Photosynthesis occurs primarily in leaves, and some photosynthesis/ respiration-related proteins respond differently to drought stress. A total of 25 DAP spots represented 17 unique photosynthesis/respiration-related proteins of which 20 and 16 DAP spots were from CS and NC47, respectively, and 11 DAP spots were common across both cultivars. In particular, the accumulation of OEE2 (spots 64 and 65) in seedling leaves was upregulated in NC47 and CS. We found just one DAP spot, identified as ribulose-phosphate 3-epimerase (RPE, spot 34), which was upregulated under 15% and 30% PEG6000 concentrations in NC47, while it was significantly downregulated in CS. In addition, ferredoxin-NADP(H) oxidoreductase (spot 32), OEE1 (spot 44) and chlorophyll *a–b* binding protein 8 (spot 56) were downregulated in both cultivars. Ribulose bisphosphate carboxylase activase (spots 9 and 30) and photosystem I subunit VII (spot 87) were identified as DAPs in CS, while they displayed no significant changes under drought stress in NC47.

Under PEG6000-mediated drought stress, some carbon metabolism-related proteins, including cytosolic aconitase (spot 7), arabinoxylan arabinofuranohydrolase (spot 15), NADP-dependent malic enzyme (spot 19), glyceraldehyde 3-phosphate dehydrogenase isoenzyme (GAPDH, spot 45) and triosephosphate isomerase (TPI, spot 97), were dramatically upregulated in NC47 and CS. Chloroplastic aldolase (spot 27) and chloroplast fructose-bisphosphate aldolase (spot 40) were downregulated in both cultivars. Phosphoglycerate mutase (PGM, spot 16), enolase (ENO, spot 22), 6-phosphogluconate dehydrogenase (6PGD, spot 26) and beta-amylase (spot 90) were significantly upregulated in NC47, but downregulated or unchanged in CS in response to increased drought stress. Expression levels of PGM, ENO and 6PGD were significantly higher in NC47 than in CS.

Wheat seedlings are always prone to drought stress in natural environments. Some stress/defence/detoxification-related proteins, including ROS scavengers, were affected significantly. We identified 11 and 14 DAP spots from CS and NC47, respectively, and 8 DAP spots common to both cultivars. Among these DAPs, dehydroascorbate reductase (DHAR, spot 61), ascorbate peroxidase (APX, spot 62), Cu/Zn superoxide dismutase (SOD, spot 73), 2-Cys peroxiredoxin (spot 76) and a fibrillin-like protein (spot 95) were upregulated in NC47, but no obvious differences were observed in CS. The accumulation of 2-Cys peroxiredoxin (spot 76) and a fibrillin-like protein (spot 95) were distinctly higher in NC47 than in CS. 2-Cys peroxiredoxin is one of the peroxiredoxins (Prxs), a ubiquitous family of antioxidant proteins. Furthermore, two DAP spots (spots 48 and 50) were identified as polyamine oxidase (PAO), which was downregulated in NC47 under PEG6000-induced drought stress, but upregulated in CS.

Additional drought-response proteins also showed significantly different accumulation patterns between CS and NC47. As shown in S1 Table, methionine synthase 1 enzyme (spots 5 and 6), ATP synthase beta subunit (spot 24) and Cp31BHv (spots 80 and 81) were downregulated in both cultivars under drought stress. In addition, the vacuolar proton-ATPase subunit A (spot 14), chitinase 2 (spot 58), predicted protein (spot 63), 50S ribosomal protein L1 (spot 69), the 70-kDa heat shock protein (HSP70, spot 3) and RuBisCO large subunit-binding protein (spot 11) were upregulated in NC47, but no distinct differences were observed in CS.

### Network analysis of the key DAPs from NC47 seedling leaves involved in the drought response

**Fig 6:**
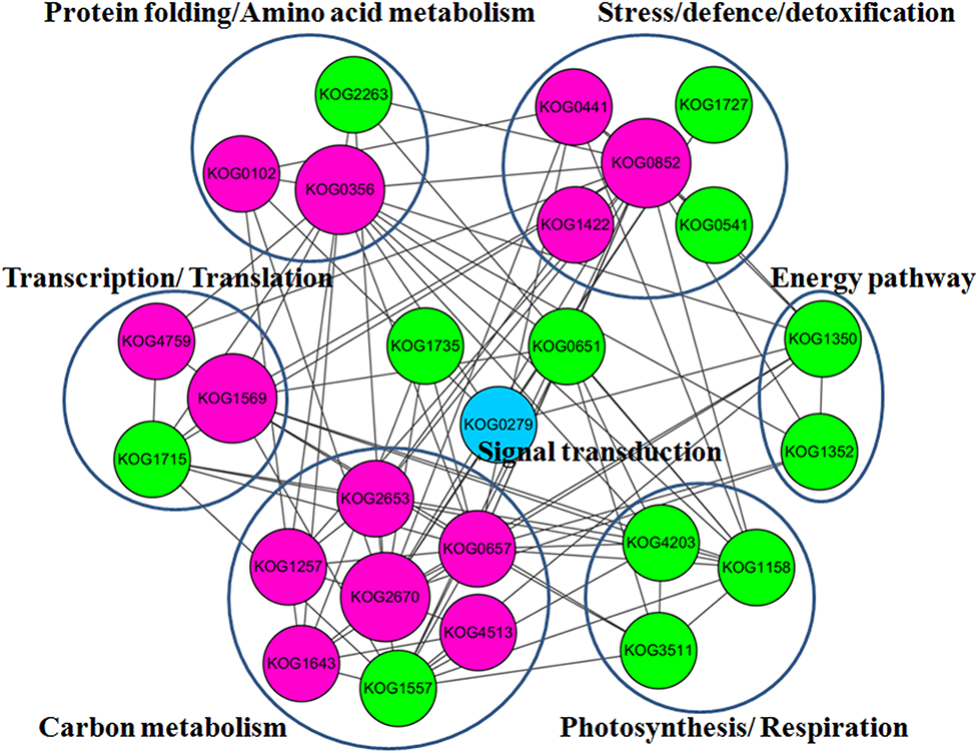
Network of key DAPs in NC47 involved in drought adaptation and tolerance. Interactions of the DAPs are extracted by searching the STRING database with a confidence cutoff of 0.700. The interaction network is reconstructed using the Cytoscape software. Red colour represents the upregulated proteins and green colour shows the downregulated proteins in NC47 under high PEG6000 concentration stress. Blue colour indicates the signal transduction-related protein.

Abbreviations: KOG0852, 2-Cys peroxiredoxin BAS1 (spot 76); KOG0441, Cu/Zn SOD (spot 73); KOG1422, dehydroascorbate reductase (spot 61); KOG0102, HSP70 (spot 3); KOG0356, RuBisCO large subunit-binding protein subunit beta (spot 11); KOG4759, predicted protein (Ribosome recycling factor, spot 63); KOG1569, 50S ribosomal protein L1 (spot 69); KOG2653, 6PGD (spot 26); KOG1257, NADP-dependent malic enzyme (spots 17 and 19); KOG2670, ENO (spot 22); KOG0657, GAPDH (spot 45); KOG1643, TPI (spot 97); KOG4513, PGM (spot 16); KOG0279, predicted protein (G protein beta subunit-like protein, spot 96).

To identify the interactions of DAPs and their potential substrates in NC47, protein–protein interaction (PPI) analysis was conducted using STRING (version 9.1). Thirty KOGs (S1 Table), representing 40 key unique DAPs, were used to construct an interaction network of the DAPs. To improve the reliability of PPI analysis, the confidence score was set at ≥0.70. The PPI network of all DAPs, with their potential substrates in NC47, was extracted from the whole interaction network and reconstructed using the Cytoscape software (Fig 6). The red colour represents the upregulated proteins and green shows the downregulated proteins in NC47 under a high PEG6000 concentration. The PPI network showed that these upregulated proteins play important roles in the different protein functional groups under drought stress. For example, enolase (KOG2670, spot 22) interacts with 19 other proteins involved in amino acid metabolism, carbon metabolism, energy pathway, signal transduction, stress/defence/detoxification, protein folding and nucleotide metabolism. Furthermore, 2-Cys peroxiredoxin BAS1 (KOG0852), RuBisCO LSBP (KOG0356), 50S ribosomal protein L1 (KOG1569), 6PGD (KOG2653), GAPDH (KOG0657) and HSP70 (KOG0102) had close interactions with other DAPs in NC47. In addition, these downregulated proteins, including a predicted protein (ribosomal protein L12, KOG1715), chloroplast fructose-bisphosphate aldolase (KOG1557), predicted protein (26S proteasome regulatory complex, KOG0651), phosphoribulokinase (KOG4203) and ferredoxin-NADP(H) oxidoreductase (KOG1158), might play an important role in the adaption to drought stress.

## Discussion

In this study, we conducted a comparative morphological, physiological and proteomic analysis of seedling leaves comparing the response of CS and NC47 to drought stress at the three-leaf stage. The morphological and physiological indices were definitely affected by drought stress. To survive drought, the cells of a wheat seedling leaf must be able to activate a defence/adaption mechanism. We identified 77 unique proteins that displayed differentially accumulated levels during the three-leaf stage under drought stress. Wheat seedling leaves have very complex responses and tolerance mechanisms under drought stress. The response of DAPs and their accumulation in drought stress are discussed below.

### Stress/defence/detoxification

In general, adverse stress is associated with imbalances in the cellular redox metabolism, resulting in an enhanced risk of oxidative damage. Therefore, an increased abundance of some ROS-scavenging enzymes has been reported in most proteomic studies focusing on abiotic stress [29]. Particularly in higher plants, antioxidant enzymes in drought-sensitive varieties are more sensitive to environmental stress than drought-tolerant varieties [10]. The generation of ROS is common during drought [30]. ROS can damage chlorophyll, protein, DNA, lipids and other important macromolecules, thus affecting plant metabolism and limiting growth and yield [31]. Therefore, plants have evolved both enzymatic and nonenzymatic systems to scavenge the ROS. Enzymes, including SOD, catalase (CAT), APX, nonspecific (guaiacol) peroxidases (PODs) and glutathione reductase, work in concert with nonenzymatic antioxidants such as glutathione and ascorbate to detoxify ROS [32].

Compared with the control groups, we found 14 unique DAPs involved in stress/defence/detoxification in response to drought stress (S1 Table). These DAPs included a fibrillin-like protein (spots 49 and 95), SODs (spots 66 and 73), APX (spot 62), DHAR (spot 61) and 2-Cys peroxiredoxin BAS1 (spot 76). These proteins were generally upregulated under high PEG6000 concentrations in NC47. According to previous research, most stress-related proteins are responsive to multiple stresses and can regulate ROS levels in defending against cell detoxification [33–34]. Within a cell, SODs constitute the first line of defence against ROS. Ascorbic acid (Asc) is a major antioxidant in plants, as it detoxifies ROS and maintains photosynthetic function. Expression of DHAR, responsible for regenerating Asc from an oxidised state, regulates the cellular Asc redox state, which in turn affects cell responsiveness and tolerance to environmental ROS [35]. A fibrillin-like protein and 2-Cys peroxiredoxin BAS1 were upregulated in NC47 under drought stress. Prxs represent a ubiquitous family of antioxidant proteins and can be divided into three classes: typical 2-Cys, atypical 2-Cys and 1-Cys Prxs. In this family of enzymes, 2-Cys Prxs have been proposed to play a key role as antioxidants in regulating the level of hydrogen peroxide (H_2_O_2_) for signal transduction [36]. ROS were reduced by chloroplast-localised detoxification mechanisms, one of which involves 2-Cys Prxs [37]. Compared to the phosphoproteome of NC47 recently reported by our lab [20], 2-Cys peroxiredoxin BAS1 was phosphorylated, suggesting the potential role of protein phosphorylation in response to adverse environmental conditions.

In addition, programmed cell death plays a critical role in the hypersensitive response in the plant defence system. One of the components that triggers it is H_2_O_2_, which is generated through multiple pathways. One example is proposed to be polyamine (PA) oxidation [38]. In this study, we found two PA oxidases (spots 48 and 50) present in both CS and NC47. PAs are small aliphatic amines implicated in a wide range of environmental stresses [39–41]. Drought stress induces changes in PA titres, which broadly correlate with drought-tolerance traits [42–43]. Therefore, these upregulated proteins, such as 2-Cys Prxs, might strengthen the tolerance of NC47 response to ROS, which is dramatically induced by drought stress.

### Photosynthesis/respiration

Environmental stresses have a direct impact on the photosynthetic apparatus, essentially by disrupting all major components of photosynthesis including the thylakoid electron transport, the carbon reduction cycle and the stomatal control of the CO_2_ supply, together with an increased accumulation of carbohydrates, peroxidative destruction of lipids and disturbance of the water balance [44]. Therefore, the accumulation patterns for most photosynthesis-related proteins are complex under drought conditions. In general, RuBisCOs contain a central core composed of four large subunit dimers, as well as eight additional small subunits; the small subunits are nuclear-encoded, but those for the large subunit are chloroplast-encoded. The synthesis and assembly of the subunits are complex and involve molecular chaperones and posttranslational modifications [45].

In this study, we found five RuBisCO-related proteins that were downregulated (spots 1 and 86) or were not different (spots 20, 83 and 85) in NC47. Under stress conditions, Rubisco clearly undergoes the downregulation of protein synthesis or degradation [46]. According to Zhao et al. [47], RuBisCO fragments display different accumulation pattern during growth. The reduction in RuBisCO content in seedlings caused photosynthetic downregulation. Furthermore, evidence suggests [48] that the regeneration of RuBisCO could be maintained by the enhanced accumulation of phosphoribulokinase (spot 93), which catalyses the phosphorylation of ribulose-5-phosphate to RuBP, a key step in the Calvin cycle for CO_2_ assimilation. In contrast, downregulation of ribulose-phosphate 3-epimerase (spots 34 and 60) contributes to diminished photosynthetic activity during drought. In addition, RuBisCO activase uses the energy from ATP hydrolysis to remove tight binding inhibitors from RuBisCO, thus playing a key role in regulating photosynthesis in plants [49]. We found two ribulose-bisphosphate carboxylase activases (spots 9 and 30) that had no distinct difference in NC47, but were downregulated in CS. The function of carboxylase was restrained, while the function of oxygenase was improved or enhanced under drought stress, thus enhancing the respiration of the plant. Drought stress reduced photosynthesis and enhanced the respiration of wheat seedlings. Furthermore, oxygen-evolving enhancer proteins (OEEs) are key enzymes in the photosynthesis system, and the accumulation of OEE2 was enhanced in response to stress induced by salt and ABA [50]. In our experiment, we detected two OEE2 (spots 64 and 65) that were upregulated in two wheat cultivars (Fig 5).

### Carbohydrate metabolism

Plant carbohydrate metabolism is often deleteriously affected by drought stress. In this study, we identified some key enzymes, such as phosphoglycerate mutase (spot 16), enolase (spot 22), NADP-dependent malic enzyme (spots 17 and 19) and cytosolic aconitase (spot 7), which are involved in glycolysis and the tricarboxylic acid cycle (TCA) and upregulated under drought stress. These enzymes change in response to drought [34,51–52]. Among these enzymes, enolase was upregulated in NC47 under drought stress, while it showed no obvious difference in CS. In addition, glycolysis and the TCA provide not only energy and cofactors, but also some important substrates for the synthesis of metabolites or signals for feedback [33]. Under salt stress, Nam et al. [53] and Amini et al. [54] found that the expression of phosphoglycerate mutase was elevated in transgenic rice (*Oryza sativa* L.) and tomato (*Solanum lycopersicum* L.) tissue. Previous studies reported that the accumulation of enolase was upregulated in different varieties under drought stress and other abiotic stresses [53,55–57]. The activity of the NADP-dependent malic enzyme (NADP-ME) was typically enhanced under various stresses, including those caused by PEG, SA, cold, darkness and NaCl, and RT-PCR showed that the transcript accumulation of NADP-ME in the leaves was distinctly affected by various stresses [58]. The upregulated accumulation of cytosolic aconitase plays a role in mediating oxidative stress and regulating cell death, which might enhance the tolerance of wheat to drought stress [59]. Therefore, these upregulated proteins of carbohydrate metabolism might contribute to stronger drought tolerance of NC47 compared to CS.

### Protein folding

Under PEG6000-induced drought stress, the risk of improper protein folding increases. The category of proteins involved in protein-folding mechanisms from this study includes four proteins: HSP (spot 3), RubisCO large subunit binding protein subunit alpha (spot 10), RubisCO large subunit-binding protein subunit beta (spot 11) and peptidyl-prolyl *cis–trans* isomerase (spot 75). HSP70 and the RubisCO large subunit-binding protein subunit beta increased in response to drought in NC47 compared to the control (Cluster 2 in Fig 5A). The increase in the RubisCO large subunit-binding protein subunit beta (spot 11) protein indicates an enhanced risk of misfolding, degradation and loss of function of the key photosynthetic enzyme RubisCO under drought stress.

HSPs are known to participate in protein folding at the expense of ATP, revealing ATPase activity, and each HSP subgroup reveals a specific role in protein folding (chaperone function) and protection of nascent proteins during their transport into specific organelles such as plastids and mitochondria [29]. In our study, HSP70 (spot 3) displayed an increased accumulation in NC47 compared with control conditions or CS. In addition, an increase in HSP70 was found in various plants or various stress conditions, indicating an enhanced need for protein protection under drought stress [10,20,29]. The upregulated accumulation of HSP70 (spot 3) and RubisCO large subunit-binding protein subunit beta (spot 11) could enhance the tolerance of NC47 response to drought stress.

### Other metabolisms

Other proteins associated with energy pathway, amino acid metabolism and transcription/translation also play indispensible roles in response to drought stress. Previous studies showed that stress conditions, such as drought and salt stresses, induce V-ATPase to exhibit high flexibility and plasticity, which are essential for plant survival [60–61]. The research showed that the activity of V-ATPase was linked to salt tolerance in several species, such as sunflower (*Helianthus annuus* L.) and beet (*Beta vulgaris* L.). Other reports showed an increase in the activity of this enzyme in response to aluminium stress in wheat, suggesting that increased V-ATPase activity could be required as a homeostatic mechanism to maintain the cytoplasmic pH near neutrality [62]. Our results indicated that vacuolar proton-ATPase subunit A (spot 14) was significantly upregulated in NC47 compared to CS under drought stress (Table 1). Then, vacuolar proton-ATPase subunit A consumes ATP to transfer protons into a vacuole. Through that process, the proton transmembrane gradient is produced, which provides the power to transport various ions and metabolites [63]. Therefore, the upregulated accumulation of vacuolar proton-ATPase subunit A may increase the efficiency of water utilisation or enhance the tolerance of wheat to drought stress.

Then, according to previous studies, methionine synthase catalyses reactions, resulting in the formation of methionine from which ethylene and PAs are produced [4]. In this study, we found that methionine synthase (spots 5 and 6) was downregulated in both cultivars under drought stress. Reduction in methionine under drought has implications in signalling due to its direct effect on ethylene and PAs, and could cause changes in lignification of the cell wall through decreased methylation of lignin monomers, which may be one of the mechanisms by which cell growth is suppressed during drought stress [4]. In addition, leucine aminopeptidases were observed to be affected by drought stress, and upregulated in NC47 and downregulated in CS. In plants, aminopeptidases modulate wound signalling, meiotic recombination, cell cycle progression and embryonic and seedling development [64]. Therefore, the upregulated leucine aminopeptidases might also contribute to the stronger drought tolerance of NC47.

In addition, cp31BHv is a chloroplast RNA-binding protein, which was thought to be associated with the chloroplast ribosomal complex and correlated with the stage of leaf development [65]. Then, we discovered that cp31BHv (spots 80 and 81) was downregulated in both cultivars under drought stress. This change may influence structure formation and energy metabolism of chloroplasts in wheat seedling leaves, and may be one of the mechanisms by which leaf growth is suppressed during drought stress (Fig 1A).

### A putative pathway of proteomic metabolic changes in wheat (NC47) seedling leaf under drought stress

**Fig 7:**
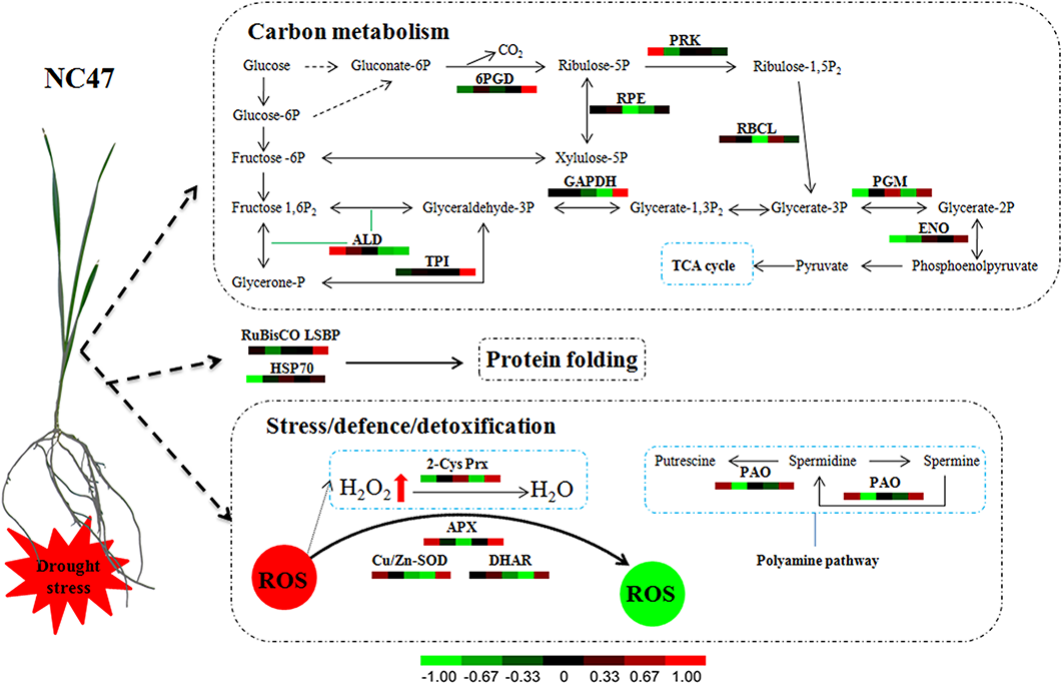
Drought stress-mediated changes in metabolic pathways of NC47 seedling leaves.

Abbreviations: ALD, fructose-bisphosphate aldolase (chloroplast); APX, ascorbate peroxidase; Cu/Zn-SOD, Cu/Zn superoxide dismutase; DHAR, dehydroascorbate reductase; ENO, enolase; 6PGD, 6-phosphogluconate dehydrogenase; GAPDH, glyceraldehyde 3-phosphate dehydrogenase; HSP70, 70-kDa heat shock protein; PAO, polyamine oxidase; PGM, phosphoglycerate mutase; PRK, phosphoribulokinase; RBCL, ribulose-1,5-bisphosphate carboxylase large subunit; ROS, reactive oxygen species; RPE, ribulose-phosphate 3-epimerase; RuBisCO LSBP, RuBisCO large subunit-binding protein; TPI, triosephosphate isomerase; 2-Cys Prx, 2-Cys peroxiredoxin BAS1.

Although different features of plant response to drought stress are documented, information of drought-tolerance mechanisms of wheat seedling leaves at the proteome level is limited [67]. Therefore, we proposed a putative metabolic pathway for the response of wheat seedling leaves to drought stress at the proteomic level. Results from our study and previous research could provide an overview of the drought-response mechanism (Fig 7). When wheat seedlings encounter drought stress, the signals of osmotic stress are transmitted from roots to leaves, which activate the ROS scavenging system in seedling tissues. These stress/defence/detoxification-related proteins, including APX, Cu/Zn SOD, DHAR, PAO and 2-Cys Prx, are upregulated. They protect wheat seedling leaves from ROS damage, and the changes in PAO and 2-Cys Prx accumulation further affect PA and H_2_O_2_ metabolism. In addition, drought stress induces some key protein folding-related proteins, such as HSP70 and RubisCO large subunit-binding protein, which were significantly upregulated. So some unknown proteins might interact with HSP70 and probably function as a molecular chaperone under drought stress. Since the main physiological function of leaves is photosynthesis and carbon metabolism, the accumulation of DAPs involved in photosynthesis and carbon metabolism is significantly altered under drought stress, which distinctly affects carbon metabolism at the wheat seedling stage. Proteomic findings of drought-stressed wheat indicate that DAPs associated with stress/defence/detoxification, protein folding, carbon metabolism, transcription/translation and signal transduction play key roles in drought adaptation and tolerance.

## Conclusions

In this study, we performed an in-depth analysis of the physiological and proteomic changes of wheat seedling leaves under PEG6000-induced stress with two spring wheat cultivars: drought-tolerant NC47 and drought-sensitive CS. Seventy-seven unique proteins responding to drought stress were identified by 2-DE and MALDI-TOF-MS. These proteins included 101 DAP spots, which were categorised into 10 groups according to their putative functions. Some key DAPs such as enolase, 6-phosphogluconate dehydrogenase, OEE2, fibrillin-like protein, 2-Cys peroxiredoxin BAS1 and HSP70 were dramatically upregulated in NC47, and they played important roles in response to drought stress, which could contribute to the stronger drought tolerance of NC47 compared to CS. Our comprehensive proteomic results provide an additional perspective on seedling development and growth responses to drought stress. Differential accumulation proteins, identified for the two spring wheat cultivars under normal and drought stress conditions, could be used in future studies focusing on the tolerance of wheat to drought stress.

## Supporting Information

S1 Fig 2-DE maps of all PEG6000 treatments in NC47 and CS. ‘Chinese Spring’ (CS) and ‘Ningchun 47’ (NC47), during 48 h of PEG-mediated drought stress. 0, 15%, 20%, 25% and 30% represent the PEG6000 concentration.
(TIF)

S1 Table Complete list of 101 differentially accumulated proteins in NC47 and CS seedling leaves. a) Spot number as given in Fig 2. b) Accession number: according to the NCBI database. c) Protein Score: statistical probability of true positive identification of the predicted protein calculated by MASCOT with 0.3 peptide tolerance and one allowed missed cleavage. d) Protein Score C.I.%: the PMF score percentage of protein sequence (Confidence interval: Protein Score C.I.% ≥ 95%). e) Pep. Count: matched peptide count. f) TpI/TMW (kDa): pI of predicted protein/molecular mass of predicted protein. g) Cyto, cytoplasm; P: plastid; Mito: mitochondria; Nucl: nuclear; PM: Plasma membrane. h)P-value with three times repeat. i) KOG/NOG number searched by eggNOG. "N" represent the DAP spot was no obvious difference (< 2.0-fold), "—" represent the DAP spot was none.
(XLS)

S2 Table The peptide sequences of all DAPs. (XLS)
