# Supplementary figures and images for: Identification of Leaf Proteins Differentially Accumulated between Wheat Cultivars Distinct in Their Levels of Drought Tolerance
Source: PLoS One. 2015 May 18;10(5):e0125302. doi: 10.1371/journal.pone.0125302 (PMC4436182; doi:10.1371/journal.pone.0125302)

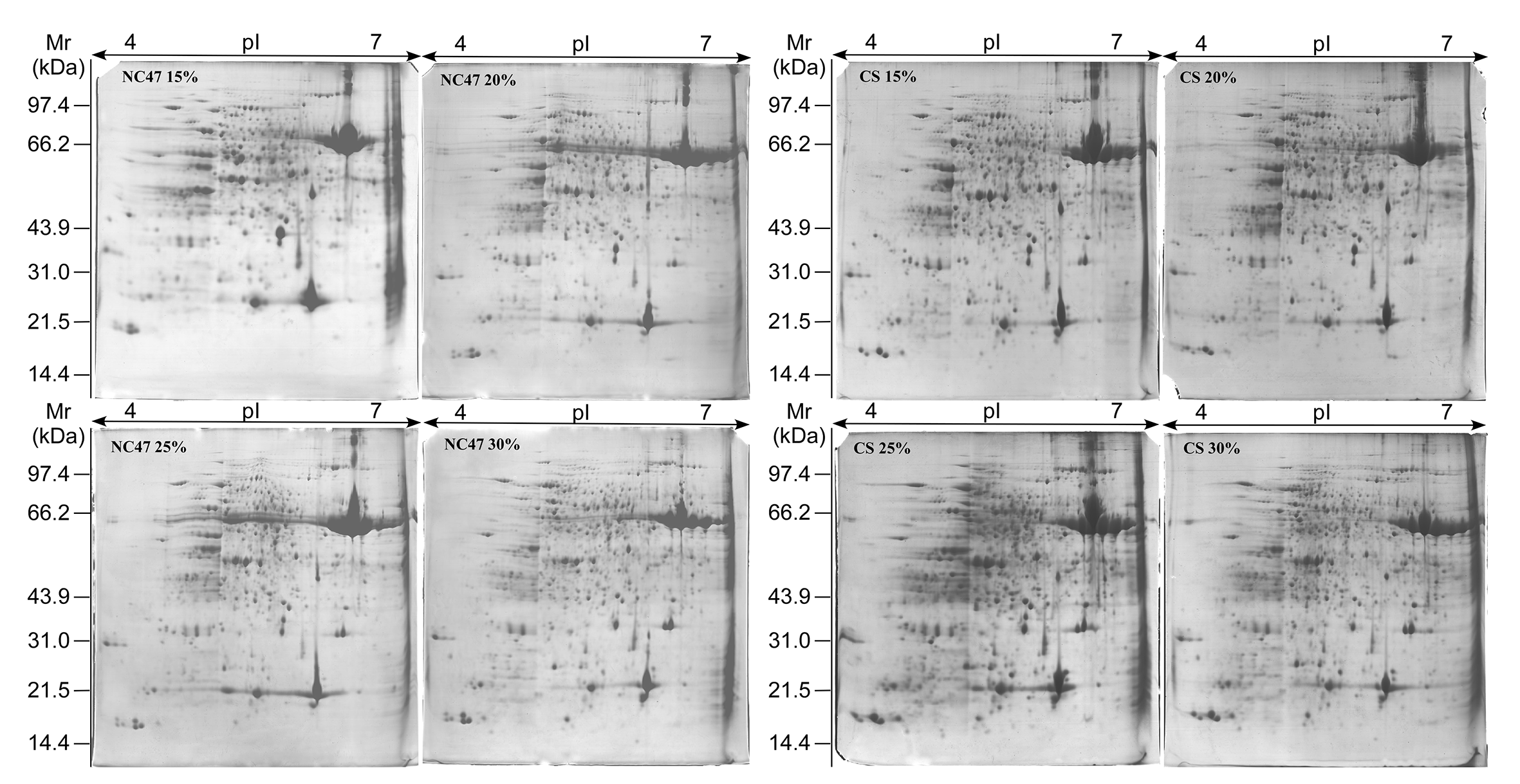

Supplement: S1 Fig — ‘Chinese Spring’ (CS) and ‘Ningchun 47’ (NC47), during 48 h of PEG-mediated drought stress. 0, 15%, 20%, 25% and 30% represent the PEG6000 concentration. (TIF) [file pone.0125302.s001.tif]
